# Publisher Correction: Light-driven formation of manganese oxide by today’s photosystem II supports evolutionarily ancient manganese-oxidizing photosynthesis

**DOI:** 10.1038/s41467-020-20868-9

**Published:** 2021-01-12

**Authors:** Petko Chernev, Sophie Fischer, Jutta Hoffmann, Nicholas Oliver, Ricardo Assunção, Boram Yu, Robert L. Burnap, Ivelina Zaharieva, Dennis J. Nürnberg, Michael Haumann, Holger Dau

**Affiliations:** 1 Physics Department, Freie Universität Berlin, Arnimallee 14, 14195 Berlin, Germany; 2 Department of Microbiology and Molecular Genetics, Oklahoma State University, Stillwater, OK 74078-4034 USA; 3 Present Address: Department of Chemistry - Ångström Laboratory, Molecular Biomimetics, Uppsala University, Lägerhyddsvägen 1, 75120 Uppsala, Sweden

**Keywords:** Metals, Origin of life, Molecular evolution, Photosystem II, Biogeochemistry

Correction to: *Nature Communications* 10.1038/s41467-020-19852-0, published online 30 November 2020.

The original HTML version of this Article was updated shortly after publication because the previous Peer Review file was mistakenly labelled as the Source Data file, and the Source Data file was mistakenly labelled as Peer Review file.

