# Supplementary material for: Light-driven formation of manganese oxide by today’s photosystem II supports evolutionarily ancient manganese-oxidizing photosynthesis
Source: Nat Commun. 2020 Nov 30;11:6110. doi: 10.1038/s41467-020-19852-0 (PMC7705724; doi:10.1038/s41467-020-19852-0)
Supplement: Supplementary file 1 — Suplementary Information [file 41467_2020_19852_MOESM1_ESM.pdf]

Supplementary Information

Light-driven formation of manganese oxide by today's photosystem-II supports evolutionarily ancient manganese-oxidizing photosynthesis

Petko Chernev,[1,2] Sophie Fischer,[1] Jutta Hoffmann,[1] Nicholas Oliver,[1] Ricardo Assunção,[1] Boram Yu,[1] Robert L. Burnap,[3] Ivelina Zaharieva,[1] Dennis J. Nürnberg,[1] Michael Haumann,[1] Holger Dau[1*]

[1]Physics Department, Freie Universität Berlin, Arnimallee 14, 14195 Berlin, Germany

[2]present address: Department of Chemistry - Ångström Laboratory, Molecular Biomimetics, Uppsala University, Lägerhyddsvägen 1, 75120 Uppsala, Sweden

[3]Dept. of Microbiology and Molecular Genetics, Oklahoma State University, Stillwater, Oklahoma 74078-4034, United States

Correspondence to: Holger Dau, Email: holger.dau@fu-berlin.de

This PDF file includes:

Supplementary Figures 1 to 16

Supplementary Tables 1 and 2

Supplementary Note providing background information

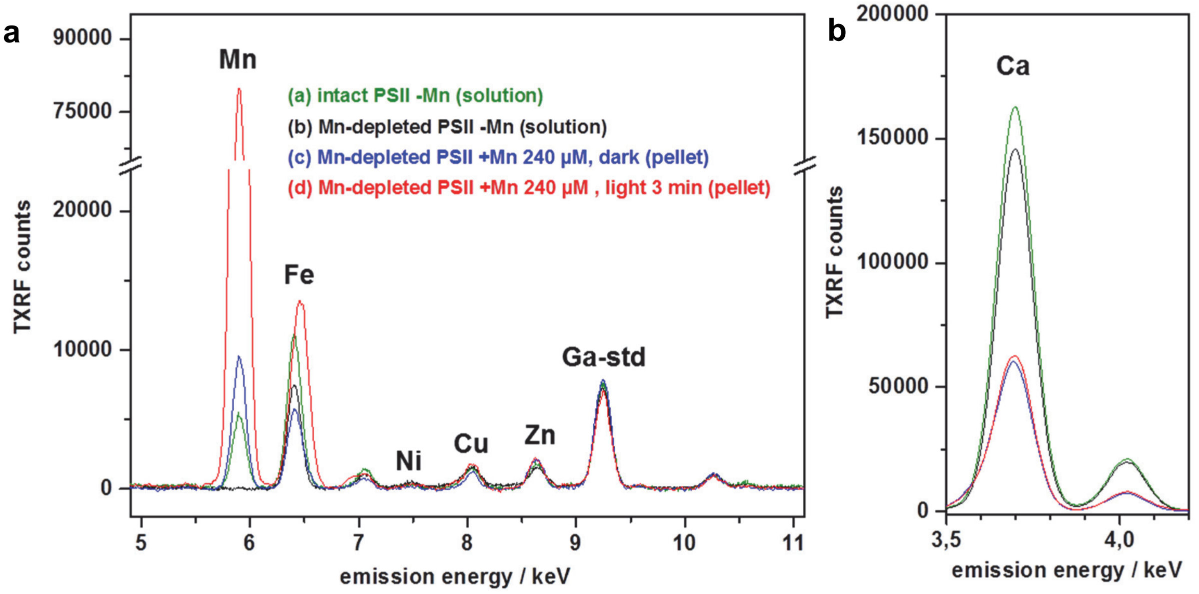

Supplementary Figure 1. Metal content determination in PSII preparations by total TXRF. A gallium concentration standard (Ga-std, 1 mg/L) was added to solutions of PSII membranes containing 1 mg/mL (~1 mM) chlorophyll (chl). Kα X-ray emission lines are labelled by element (respective unlabeled bands represent Kβ emission lines). +/-Mn denotes the presence or absence of additionally added manganese ($MnCl_2$). Respective Mn and Fe concentrations are given in Tables 1 and S1. **(a)** Spectra in the Mn to Ga region. The following samples were assayed: (a) As-prepared PSII membrane particles, which were active in oxygen evolution, were pelleted and adjusted by buffer (pH 7) addition to a chl concentration of 1 mM. (b) Mn-depleted PSII membrane particles, which were inactive in oxygen evolution, were pelleted and adjusted by buffer (pH 7) addition to a chl concentration of 1 mM. (c) Mn-depleted PSII membrane particles (20 µg/mL / ~20 µM chl) were incubated for 3 min in darkness at 20 °C with 240 µM $MnCl_2$ in buffer (pH 8.5), pelleted, and adjusted by buffer addition to a chl concentration of 2 mM. (d) Mn-depleted PSII membrane particles (20 µg/mL / ~20 µM chl) were incubated for 3 min at 20 °C under continuous white light illumination (1000 µE m^{-2} s^{-1}) with 240 µM $MnCl_2$ in buffer (pH 8.5), pelleted, and adjusted by buffer addition to a chl concentration of 2 mM. PSII membrane particles were pelleted by centrifugation at 50000 g for 12 min at 4 °C. **(b)** Spectra in the Ca region (spectra correspond to data in A). TXRF spectra were collected during ~10 min data acquisition (Bruker Picofox instrument) and metal contents were determined using the routines provided with the spectrometer. Note that the apparent shift of the Fe peak in (d) is due to the underlying large Kβ emission line of Mn. Spectra were normalized to the mean amplitude of the Ga standard Kα peak for comparison.

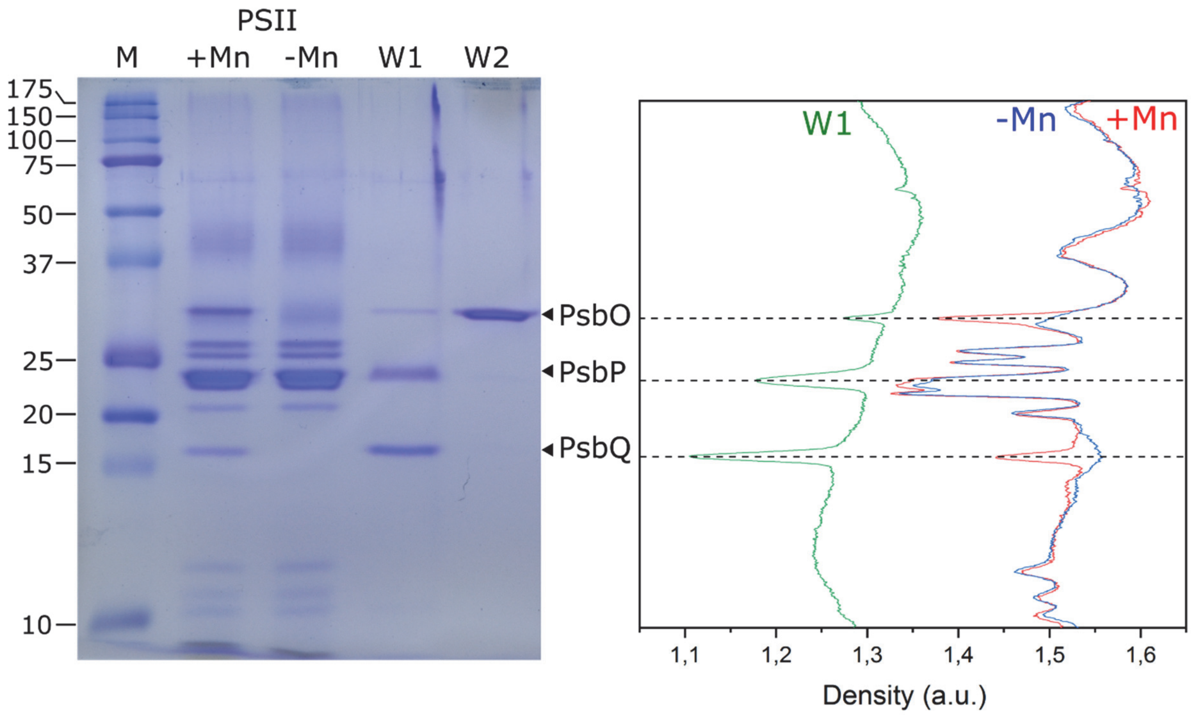

Supplementary Figure 2. SDS-PAGE of PSII-enriched membrane particles (**left panel**) and densitogram (**right panel**). The lanes in the gel shown in the left panel represent: *M*, molecular weight marker (values on the left y-axis given in kDa); *+Mn*, PSII-enriched membrane particles directly after isolation; *-Mn*, Mn-depleted PSII-enriched membrane particles lacking the extrinsic proteins PsbO (33 kDa), PsbP (24 kDa), and PsbQ (18 kDa); *W1*, supernatant after washing with high-salt buffer containing TEMED; *W2*, proteins removed by washing with high-pH buffer. PSII samples with 1 µg chlorophyll and wash samples with 1 µg protein were loaded on the gel. The data show that the Mn-depletion and washing procedures had completely removed PsbQ and mostly PsbO and PsbP from the PSII protein complex (so that they appear in the supernatant after centrifugation) in samples used for the spectroscopic experiments. This is consistent with previous results in ref.[3]. Shown data are representative for three repetitions of the gel SDS-PAGE experiment.

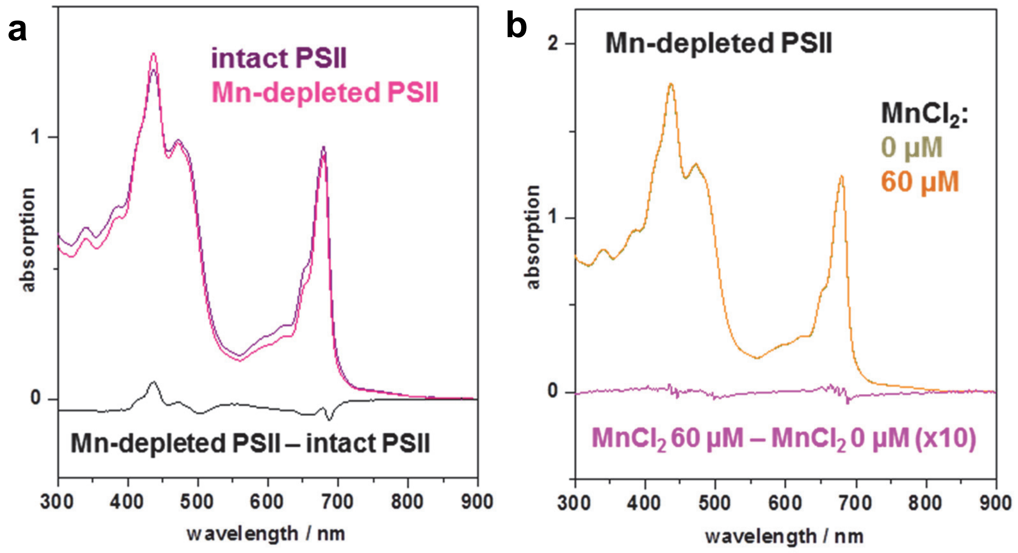

Supplementary Figure 3. Optical absorption spectra of PSII preparations. **(a)** Comparison of intact and Mn-depleted PSII membrane particles and difference spectrum. Spectral differences in Mn-depleted PSII likely reflect partial removal of light harvesting complexes from the membranes during the manganese depletion procedure involving several washing (centrifugation) steps. **(b)** Mn-depleted PSII without (0 µM) and with 60 µM $MnCl_2$ and difference spectrum. Chlorophyll 20 µg/mL, pH 7.5 in (a) and (b). $MnCl_2$ has no significant absorption in the shown spectral region (300-900 nm). The same result was obtained by control experiments at various pH values ranging from 6.2 to 8.5.

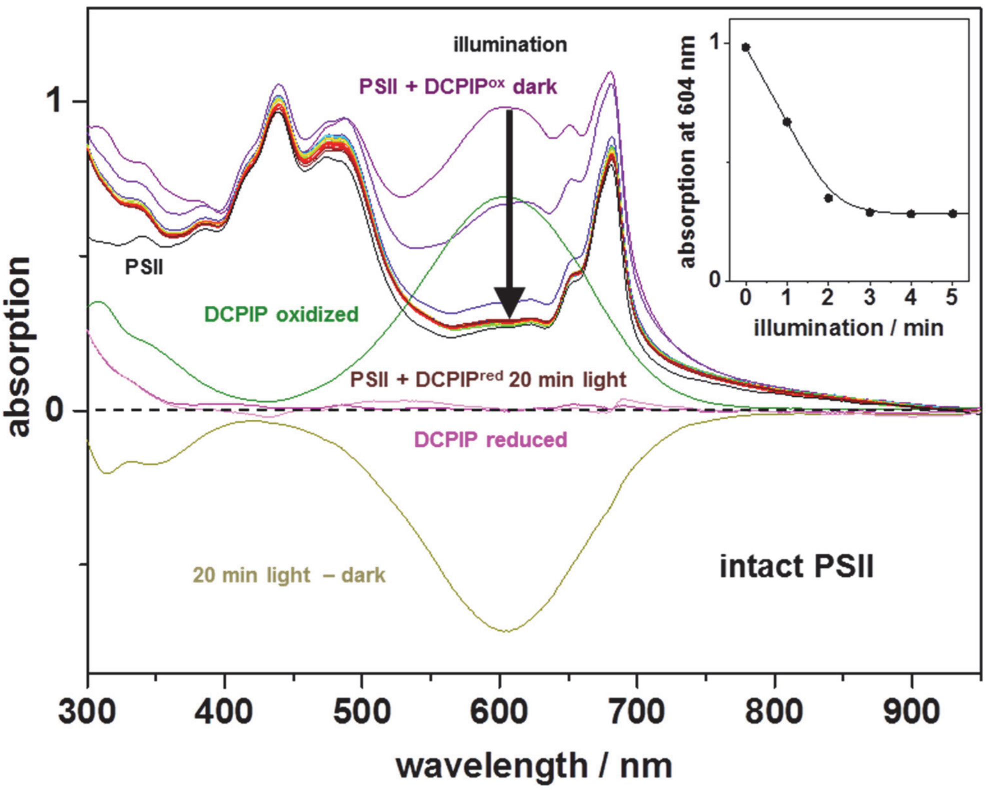

Supplementary Figure 4. Absorption spectroscopy on DCPIP reduction by intact PSII. Oxygen-evolving PSII membrane particles were illuminated with saturating white light (1000 µE m^{-2} s^{-1}, pH 7.0) and an absorption spectrum showing contributions from chlorophyll and DCPIP was recorded every 1 min. The dark spectra were obtained immediately prior to illumination. The spectrum of oxidized DCPIP (60 µM) was obtained before addition of PSII (20 µg mL^{-1} chlorophyll), the difference spectrum (20 min light – dark) shows complete DCPIP reduction, and the spectrum of reduced DCPIP (magenta line) is the difference (20 min light – dark) minus (DCPIP oxidized) or the difference (light magenta line) of (20 min light) minus (PSII). The inset shows the absorption at the maximum of oxidized DCPIP (604 nm) as function of the illumination time.

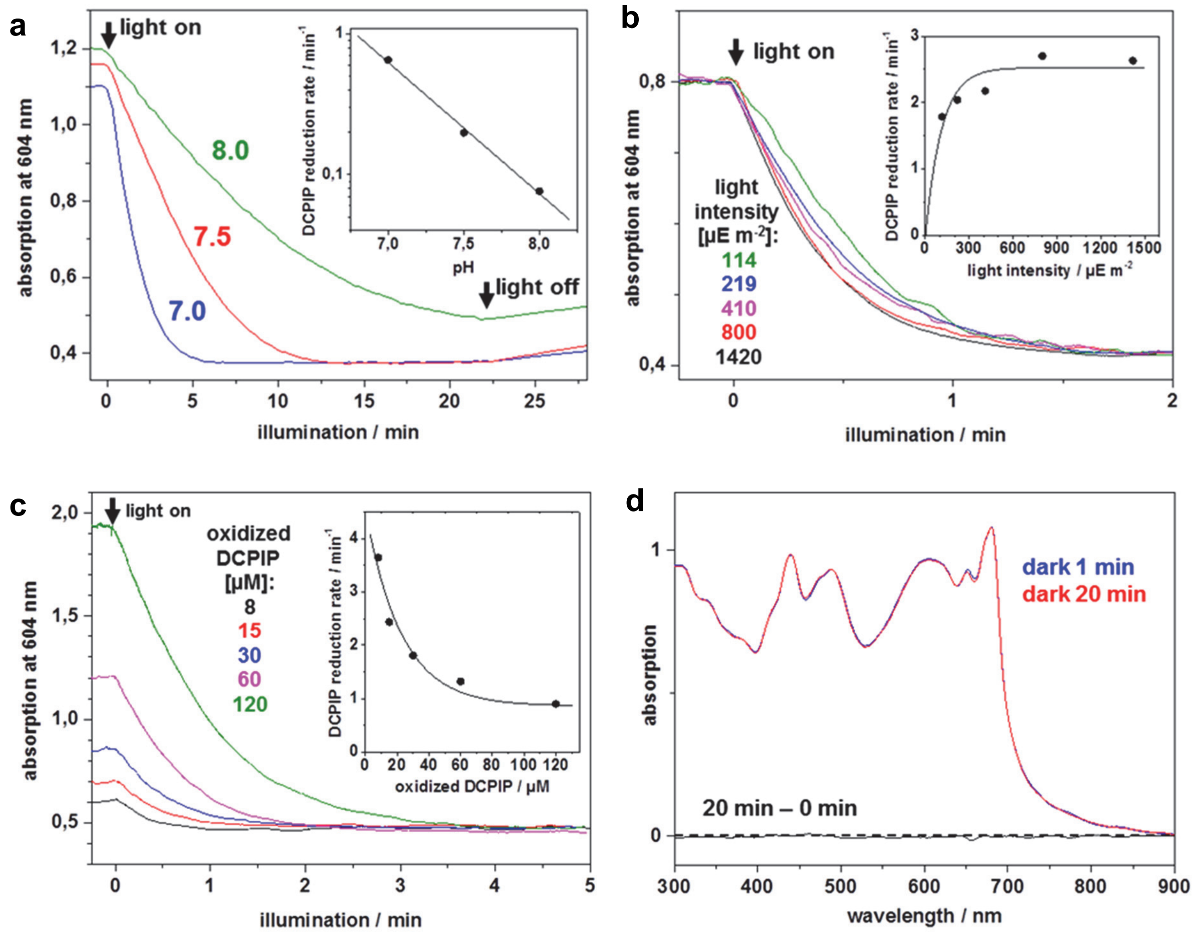

Supplementary Figure 5. Kinetics of DCPIP reduction by intact PSII. DCPIP reduction was monitored at 604 nm (PSII membrane particles, 20 µg mL^{-1} chlorophyll). **(a)** pH dependence. To oxygen-evolving PSII, 60 µM oxidized DCPIP were added in the dark and the light (1000 µE m^{-2} s^{-1}) was switched on at t = 0 min and switched off at t = 22 min (arrows). DCPIP reduction was monitored in absorption spectra as in Supplementary Figure 4. The inset shows rate constants (circles, logarithmic scale) as determined from exponential fits of transients in the main panel together with a fit curve (line). **(b)** Light intensity dependence. To intact PSII, 30 µM oxidized DCPIP was added (pH 7). The inset shows rate constants of DCPIP reduction from single-exponential fits of data in the main panel together with a saturation curve (line). **(c)** DCPIP concentration dependence. To intact PSII, oxidized DCPIP was added (pH 7) and DCPIP reduction under illumination (~1000 µE m^{-2} s^{-1}) was monitored. The inset shows rates of DCPIP reduction from exponential fits of data in the main panel together with an exponential decay curve (line). **(d)** Absence of reactions of Mn-depleted PSII in the dark. DCPIP (60 µM) and $MnCl_2$ (60 µM) were added to Mn-depleted PSII (pH 7) and spectra were collected after 1 min or 20 min darkness. No DCPIP reduction (or other changes) occurred in the dark.

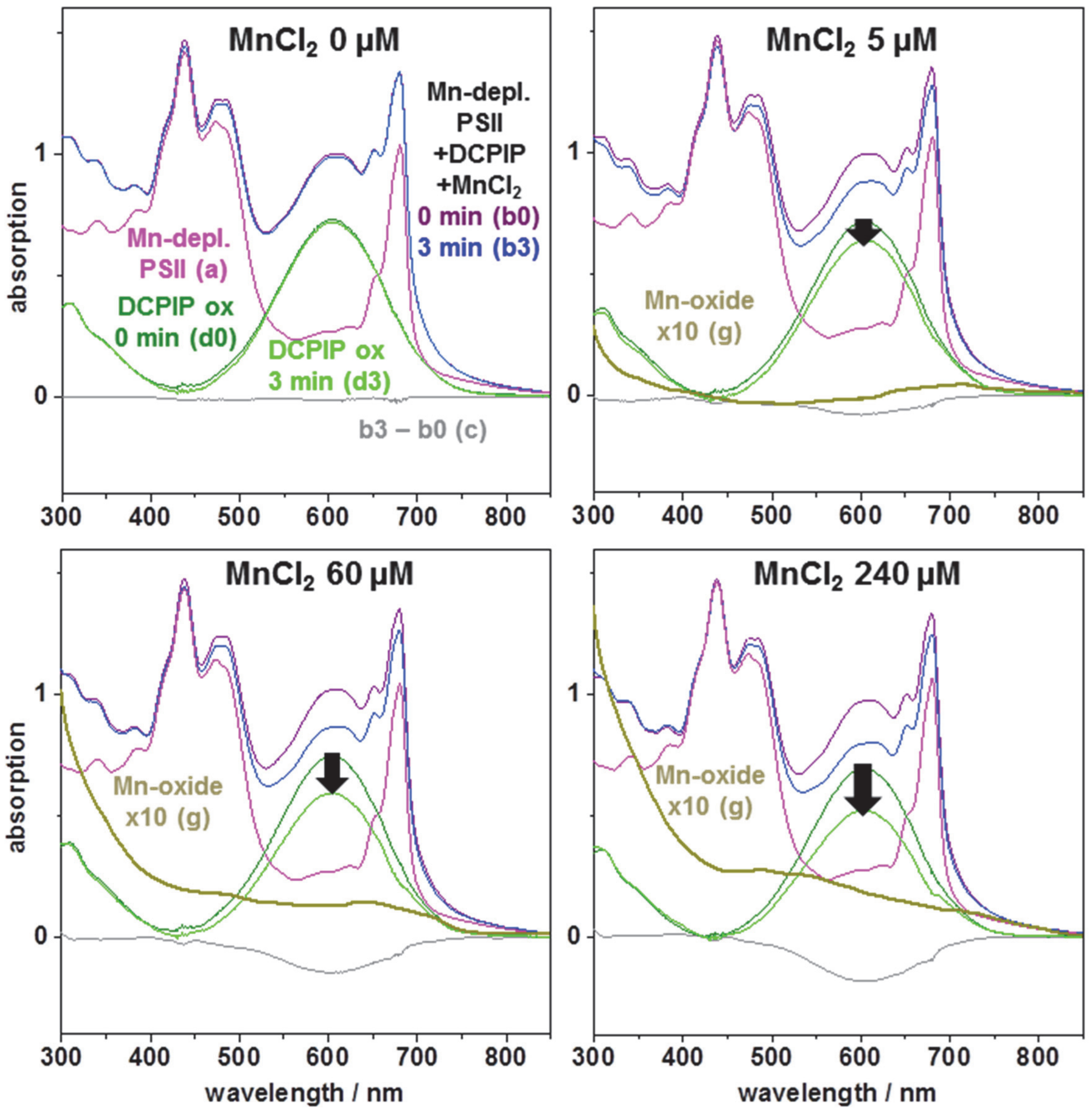

Supplementary Figure 6. Light-driven electron transfer in Mn-depleted PSII for increasing $MnCl_2$ concentrations. Shown are optical absorption spectra of PSII (magenta spectra denoted a; 20 µg mL^{-1} chlorophyll (chl), pH 7.0) to which 0-240 µM $MnCl_2$ and 60 µM $DCPIP^{ox}$ (dark-green spectra) were added (to yield spectra denoted b) and samples were illuminated with continuous white-light (1000 µE m^{-2} s^{-1}) for 0 min or 3 min (purple and blue lines; 0 min, 3 min = spectra b0, b3; see also Figure 3). The following difference spectra were calculated: c = b3 – b0 (difference after 3 min illumination); d0/3 = b0/3 – a (spectra of $DCPIP^{ox}$ after 0 min or 3 min illumination); e = c + 0.01a (0 µM $MnCl_2$; removal of chl bleaching contributions, leaving small remainders due to chl spectral changes); f = c + 0.01a + 0.10/0.20/0.27d (5/60/240 µM $MnCl_2$; chl bleaching removal and compensation for $DCPIP^{ox}$ decay); g = f – e (x10) (chl remainder removal yields spectra due to Mn oxide at 3 min illumination; smoothed by adjacent averaging of data points in a 50 nm range for clarity).

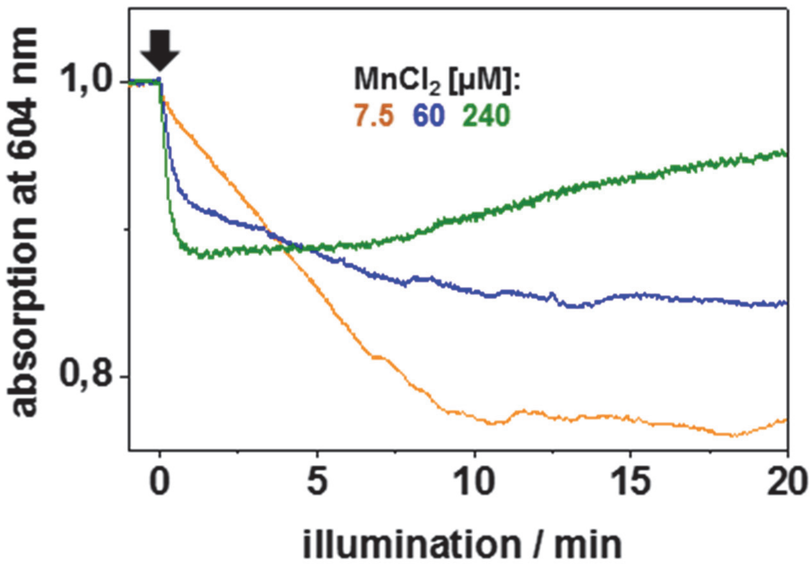

Supplementary Figure 7. Kinetics of DCPIP reduction in Mn-depleted PSII. Time traces of 604 nm absorption due to reaction of DCPIP with Mn-depleted PSII (20 µg mL^{-1} chlorophyll, pH 7.0) upon illumination (1000 µE m^{-2}, arrow) for three $MnCl_2$ contents on a longer time scale as in Figure 4. Note that DCPIP reduction continues until longer illumination times for a lower $MnCl_2$ concentration (i.e., until the oxidized acceptor is depleted). The apparent absorption increase at 240 µM $MnCl_2$ observed for longer illumination times is presumably due to slow re-oxidation of previously reduced DCPIP, possibly by O_2 in the solution or by the previously formed PSII-bound Mn oxide.

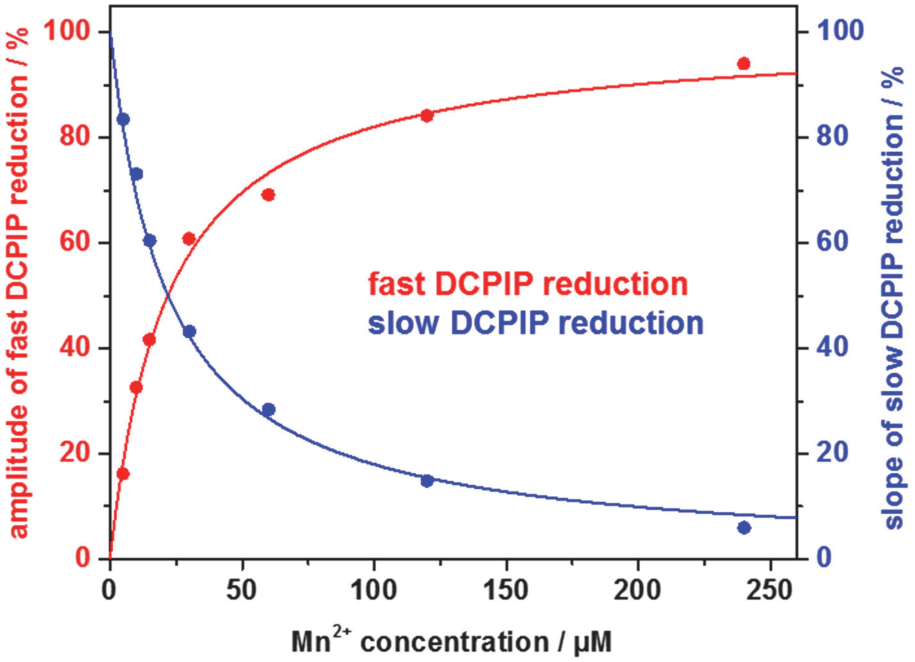

Supplementary Figure 8. Analysis of the time traces of DCPIP reduction shown in Figure 4. The amplitude of the fast absorption change, assignable to Mn oxide formation, at 604 nm after 0.75±0.05 min of illumination (red dots and line) is compared to the slope of the slow, continuous DCPIP reduction within 2-4 min of illumination (blue dots and line). The relative amplitudes of the fast reduction phase were derived by readout of the amplitude at 0.75±0.05 min in the time traces in Figure 4, subtraction of the extrapolated amplitudes at 0.75 min of a linear fit to the time traces in the range of 2-4 min and of the respective amplitude for the curve at zero Mn^{2+}, and normalization of the resulting values to the maximal fitted amplitude at high Mn concentrations (see below). The relative slopes of the slow DCPIP reduction phase were derived from a linear fit to the time traces in Figure 4 in the range of 2-4 min, subtraction of the y-axis crossing value and of the respective slope for the curve at zero Mn^{2+}, and normalization of the resulting values to the maximal fitted slope at zero Mn concentrations (see below). Data fitting with Michaels-Menten-type binding kinetics, i.e., $Y = [(Y_{max}\, c_{Mn}) / (K_m + c_{Mn})]$, showed that fast phase amplitudes and slow phase slopes were well described using the same K_m of 22±3 µM (smooth lines). Accordingly, the analysis reveals inverted behavior of the continuously ongoing DCPIP reduction and of the rapid reduction (resulting in Mn oxide formation) terminated after about 1 min of illumination. Within the respective uncertainty range, the half-effect binding constant ($K_{m,}$) for Mn oxide formation and inhibition of the continuous reduction process promoted by low Mn concentration is identical, suggesting a competitive inhibition of the latter process by formation of Mn oxide nanoparticles.

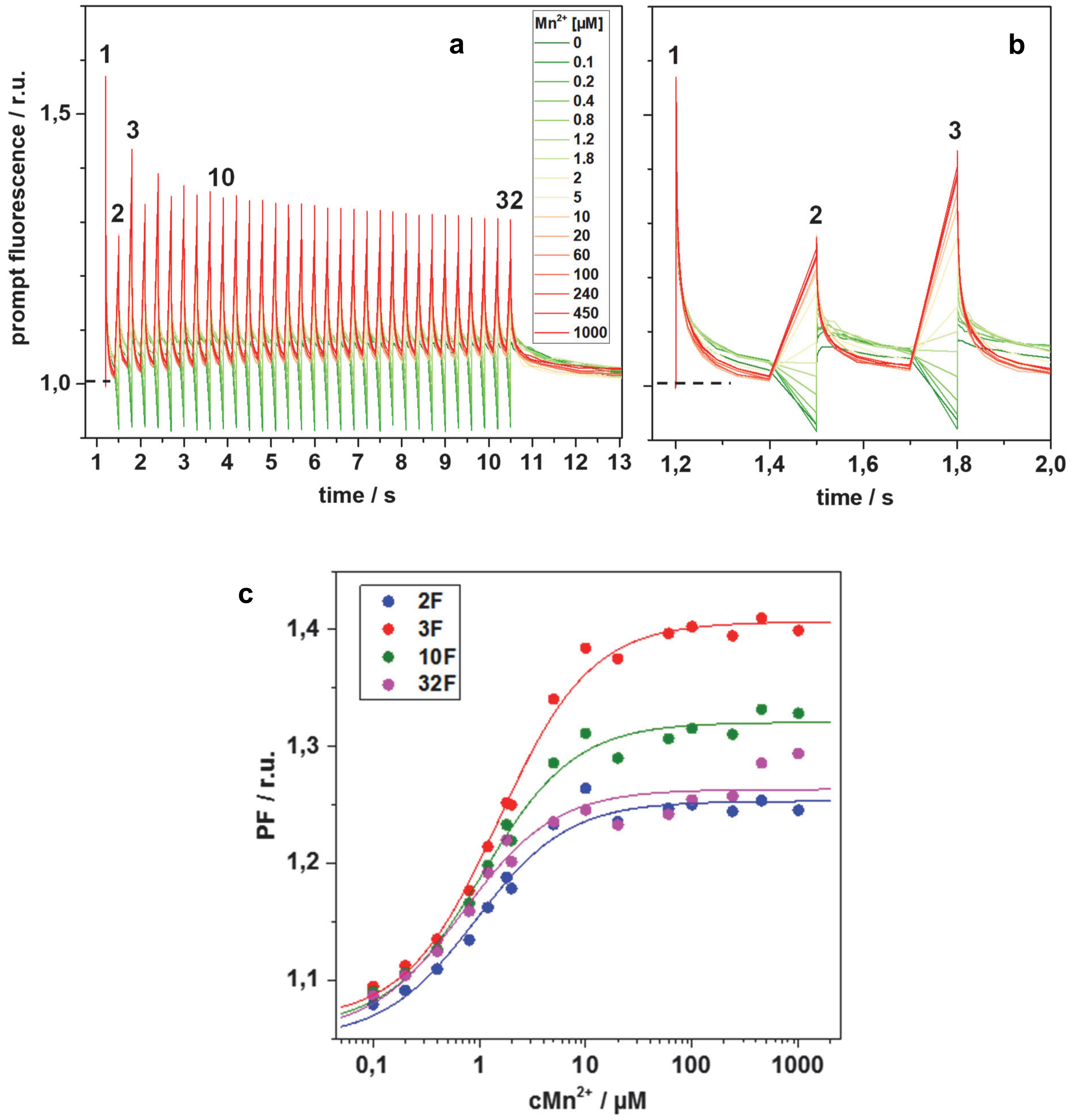

Supplementary Figure 9. Electron donation in Mn-depleted PSII facilitated by very low Mn^{2+} concentrations (K_m around 1 µM). The variable yield of the prompt fluorescence (PF) emitted by the chlorophylls of PSII [4] was measured for excitation by a train of saturating laser flashes (5 ns, 532 nm, flash spacing of 300 ms), as described elsewhere [5] (Mn-depleted PSII membrane particles, 20 µg chlorophyll per mL, 20 µM PPBQ as electron acceptor, pH 7.5). The transients were normalized to the F_0 fluorescence level recorded prior to the 1st flash (dashed line in A and B) and represent the average of 3 measurements each. The fluorescence yield was measured using weak probe flashes provided by a light-emitting diode, which were 'logarithmically' spaced in time [5]; neighboring data points are connected by straight lines (in A and B). **(a)** PF transients in a series of 32 excitation flashes at the indicated $MnCl_2$ concentrations. **(b)** PF transients for flashes 1-3. (**c**) Maximal PF amplitudes (circles) at ca. 50-500 µs after flashes 2, 3, 10, and 32 as well as fit curves (lines) assuming Michaelis-Menten-type kinetics:

PF = [(PF_{max} * c_{Mn}) / (K_m + c_{Mn})] + offset, with the following parameters (K_m/PF_{max}/offset, last-digit uncertainty range in parenthesis): 2F, 0.9(2)/0.20(1)/1.05(1); 3F, 1.5(2)/0.34(1)/1.06(1); 10F, 1.1(2)/0.26(1)/1.06(1); 32F, 0.7(2)/0.21(2)/1.05(1).

Explanation: When the first flash is applied, the redox-active tyrosine (Y_Z in Figure 1) can re-reduce the $P680^+$, the primary chlorophyll donor. At the 2nd and later flashes, however, Y_Z already is in its oxidized state prior to the flash and in the absence of an alternative electron donor, meta-stable $P680^+$ is formed upon the flash, which quenches the variable PSII fluorescence, but does not do so if binding of Mn^{2+} ions restores the electron donation capability. Therefore, at the 2nd and higher flashes, Mn-depletion results in essentially complete loss of the variable PSII fluorescence, but addition of Mn^{2+} ions results in almost complete restoration (see e.g. refs [6,7]). The above simulation results (for the panel-C data) thus can provide the Mn^{2+} binding constant for restoration of electron donation in Mn-depleted PSII. For the 2nd, 3rd, 10th and 32nd flash, the simulations result in the above binding constants with a mean value of 1.05 µM. (The period-of-two pattern visible in the fluorescence signal with restored electron donation relates to the two-electron reactions at the PSII acceptor side and is irrelevant in the present investigation.)

Context of previously published results on electron donation at low Mn^{2+} levels: Electron donation to Mn-depleted PSII at comparably low Mn^{2+} concentration has been investigated in the last 50+ years repeatedly and shown to be related to Mn binding with a pH-dependent binding constant around 1 µM (see, e.g. refs [6-12]). Some of these studies provided direct evidence for continuous electron donation, e.g. by recording the Y_Z reduction kinetics averaged over 200-400 light flashes (see e.g. refs.[11-12]). Analysis of laser-flash induced transients of the variable PSII fluorescence suggests that also for the here used PSII membrane particles, continuous electron donation is facilitated by Mn ions^{2+} with an apparent binding affinity close to 1 µM (see above). The underlying chemical process has not been convincingly clarified so far; discussed options include continuous Mn^{3+} formation [13], destructive oxidation reactions (e.g. degradations of chlorophylls or carotenoids [14]), or an alternative water-related chemistry (e.g. peroxide formation or reactions involving superoxide at the PSII acceptor side [15]).

Relation to Figure 4 – DCPIP photoreduction at low Mn^{2+} concentrations: Previously obtained results and the above analysis of the flash-induced PF transients suggest that at 5 µM $MnCl_2$ (the lowest concentration in Figure 4 of the main article), Mn^{2+} ions restore electron transfer (to $P680^+$) at the PSII donor side in most photosystems. The low rate of DCPIP reduction (small slope of respective curve in Figure 4), however, suggests involvement of a slow process, of which further chemical details are still unclear. This process facilitated by Mn^{2+} binding with a binding constant close to 1 µM likely is competitively inhibited by rapid formation of Mn oxide nanoparticles, with an apparent Mn binding constant of the latter process of 20-25 µM (Supplementary Figure 8).

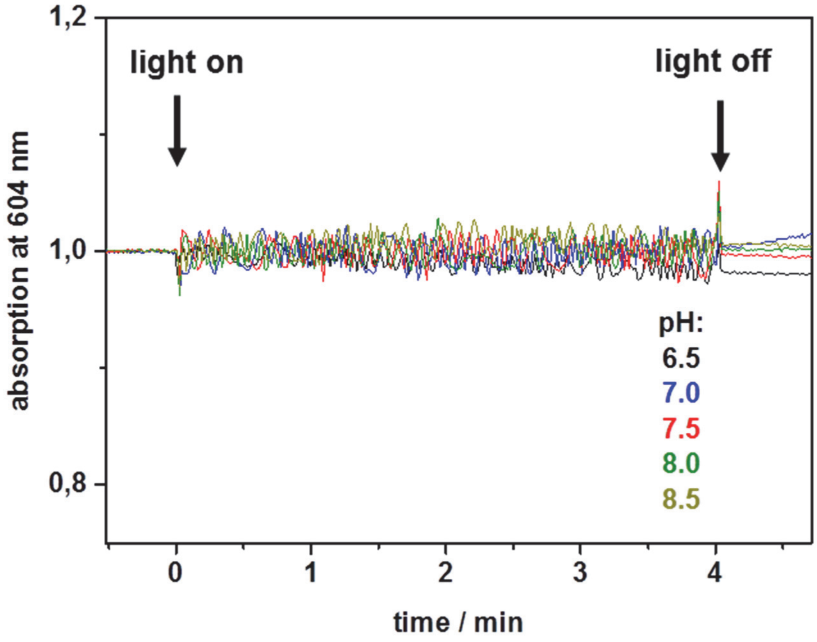

Supplementary Figure 10. Absence of DCPIP reduction without PSII. Time traces of 604 nm absorption with 60 µM DCPIP^{ox} and 240 µM $MnCl_2$ in buffers at the indicated pH values and in the absence of Mn-depleted PSII. Illumination (1000 µE m^{-2} s^{-1}) of the solutions was started or stopped at the indicated time points (arrows). Note the absence of light-induced absorption changes, which indicate the absence of reactions of $MnCl_2$ with DCPIP in the absence of PSII.

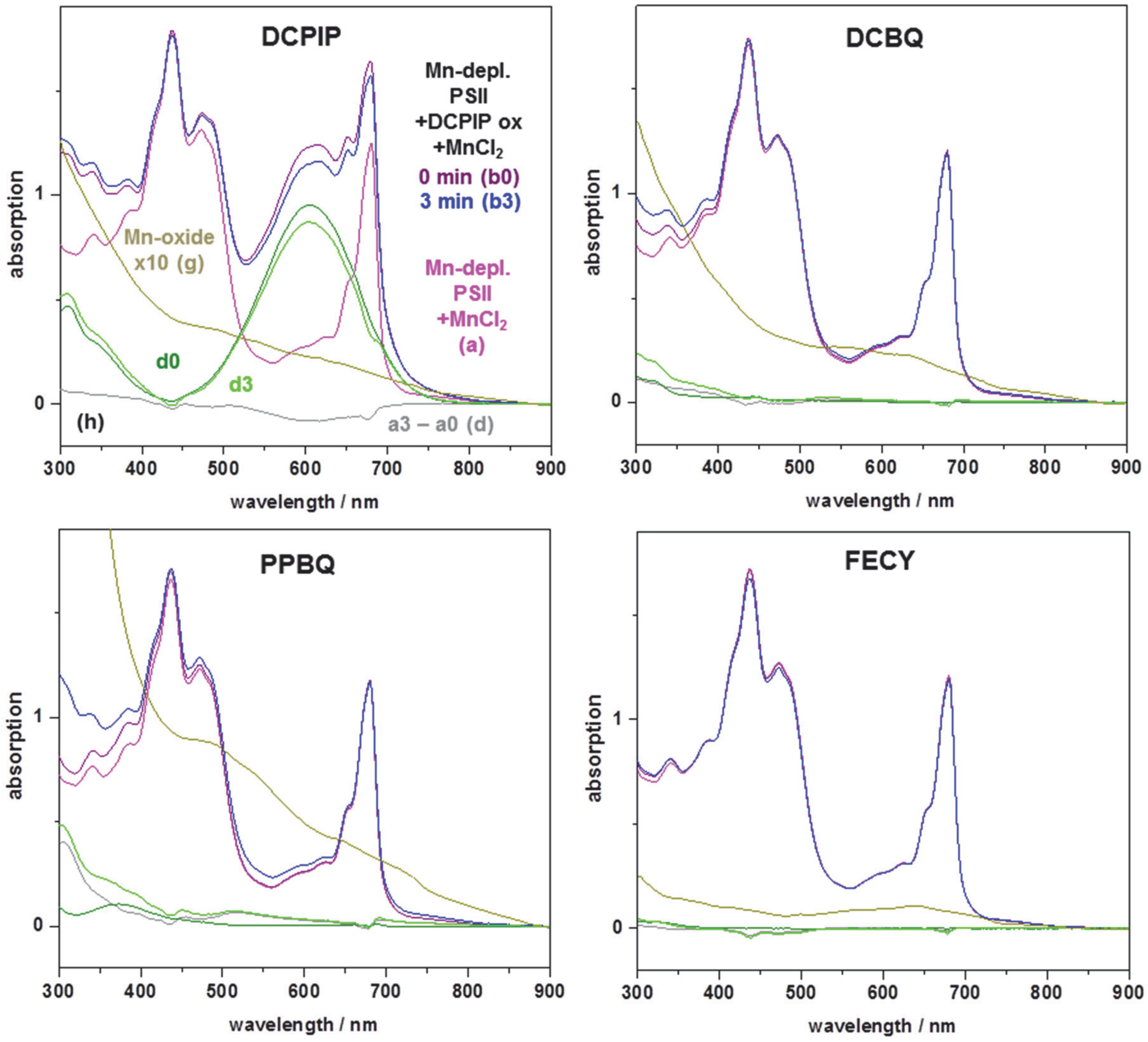

Supplementary Figure 11. Formation of Mn oxide with different electron acceptors. Electron acceptors: DCPIP = 2,6-dichlorophenol-indophenol, DCBQ = 2,5-dichloro-1,4-benzoquinone, PPBQ = phenyl-p-benzoquinone, FECY = ferricyanide ($K_3Fe(III)(CN)_6$)). Optical absorption spectra of Mn-depleted PSII (20 µg mL^{-1} chlorophyll, pH 8.5) to which 240 µM $MnCl_2$ (magenta spectra a) and initially oxidized electron acceptors (60 µM) were added and samples were illuminated with continuous white-light (4000 µE m^{-2} s^{-1}) for 0 min (dark) or 3 min (0 min or 3 min illumination = spectra b0 (purple lines) and b3 (blue lines)). Difference spectra: c = b3 – b0 (spectral differences after 3 min illumination); d = b0 – a (initial oxidized acceptors); e = c + 0.01 a (0 µM $MnCl_2$; small spectral remainders due to chl spectral changes, see Figure 3); f = c + 0.03/0.02/0.03/0.02 a + 0.09/0.10/0.28/0.05 d (DCPIP/DCBQ/PPBQ/FECY; approximate removal of chl bleaching and compensation for the decay of oxidized acceptors); g = f – e (x10) (removal of remainders results in dark-yellow spectra of reduced acceptors and oxidized manganese species after 3 min illumination; smoothed by adjacent averaging over 50 nm for clarity). We note that the use of DCPIP as a relatively hydrophilic electron acceptor supports Mn-oxide formation similarly well as the hydrophobic acceptor DCBQ. This finding is evidence that a hydrophobic acceptor is not essential for Mn-oxide formation. The different amounts of Mn-oxide formed with the different acceptors likely reflect their particular properties, especially the rate of accepting electrons at the PSII donor side and the (unwanted) ability to react with the already formed Mn oxides, but most likely not their direct involvement in oxide formation.

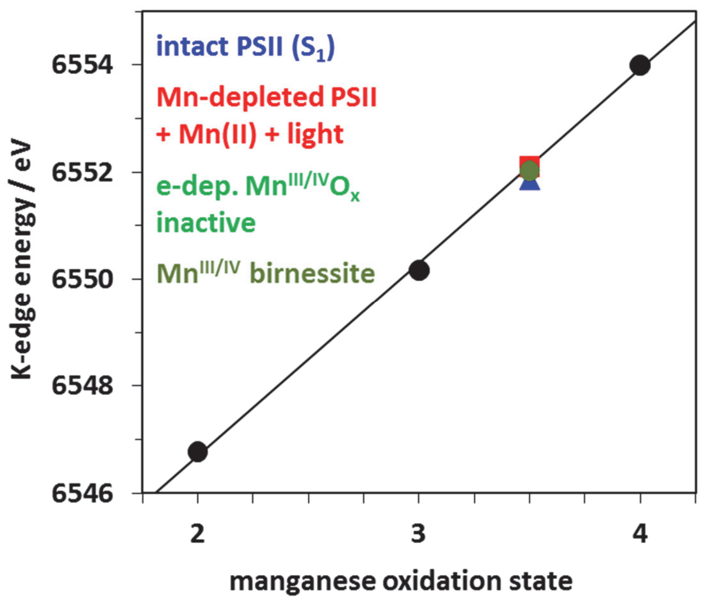

Supplementary Figure 12. K-edge energies from XANES spectra vs. manganese oxidation state. Data points stem from K-edge spectra in Figure 5A (e-dep. = electrodeposition) and were determined using the "integral method" for K-edge energy determination [16]. The line shows a linear fit (E_{edge} = 6539.5 eV + 3.6 Mn_{ox}, Mn_{ox} = Mn oxidation state) to the data points for the reference compounds ($Mn^{II}O$, α-$Mn^{III}{}_2O_3$, β-$Mn^{IV}O_2$). Note that K-edge energies of intact and Mn-depleted PSII correspond to a mean manganese redox level of +3.5, reflecting equal amounts of Mn(III) and Mn(IV) species.

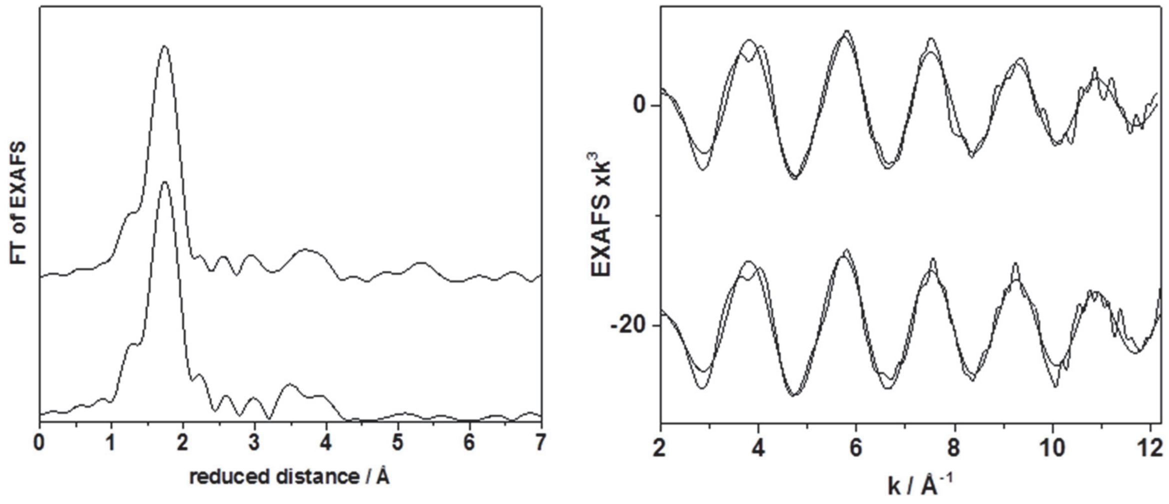

Supplementary Figure 13. Mn K-edge X-ray absorption control experiment to explore spontaneous Mn oxide formation in the absence of PSII, at an eight-fold higher Mn^{2+} concentration as used in the experiments with PSII (2 mM $MnCl_2$ dissolved in the aerobic standard buffer at pH 8 (top) and pH 7 (bottom); 60 µM PPBQ). The typical EXAFS spectra of hexaquo Mn^{2+} ions were obtained (6 Mn-O distances of ~2.15 Å); there are no indications for any spontaneous Mn oxide formation. Spontaneous Mn oxide formation was observed at pH 13. The **left panel** shows Fourier-transforms (experimental data) of EXAFS spectra in the **right panel** (vertically stacked for clarity); experimental data and simulations (smooth lines) are shown in the right panel. The resulting oxide was highly disordered and not of the birnessite-type (not shown).

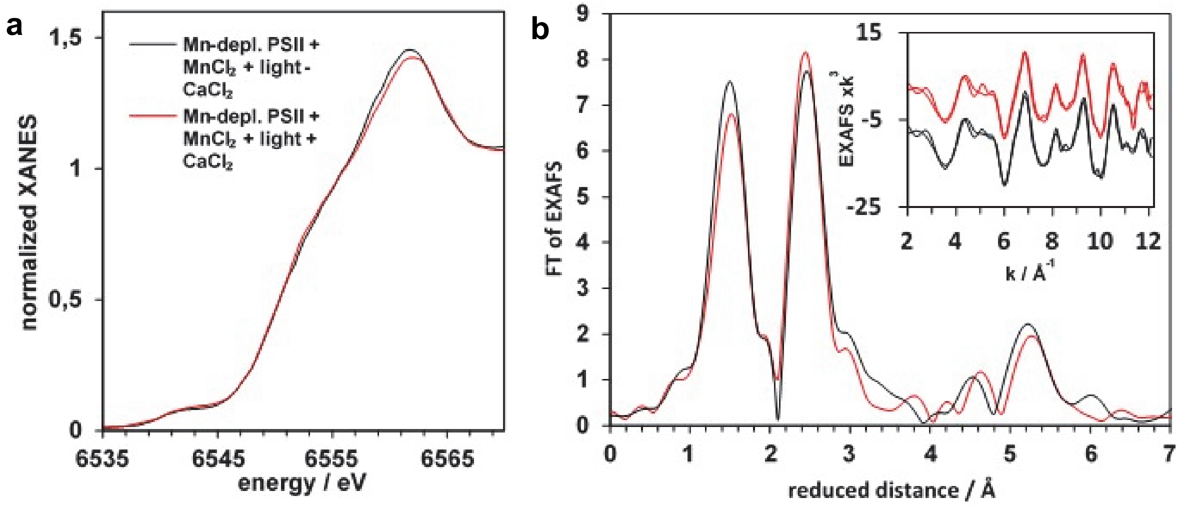

Supplementary Figure 14. XAS spectra of PSII-bound Mn oxide formed in the presence and absence of $CaCl_2$ during light-induced Mn oxide formation. **(a)** Mn K-edge XANES. **(b)** Mn K-edge EXAFS. Mn-depleted PSII samples were illuminated and XAS samples were prepared at pH 7.0 under otherwise similar conditions as used for data in Figure 5 (pH 8.5). Main panel: Fourier transforms of spectra (experimental data). Inset panel: EXAFS spectra in k-space (vertically shifted for clarity; thick lines, experimental data; thin lines, simulations with parameters detailed in Supplementary Table 2). The illumination buffer contained 240 µM $MnCl_2$ and either 5 mM externally supplied $CaCl_2$ (red spectra) or no externally supplied $CaCl_2$, herein referred to as Ca-free buffer (black spectra). For the latter experiment, $CaCl_2$ had been removed from the suspension of Mn-depleted PSII by two rounds of centrifugation and resuspension steps in Ca-free buffer.

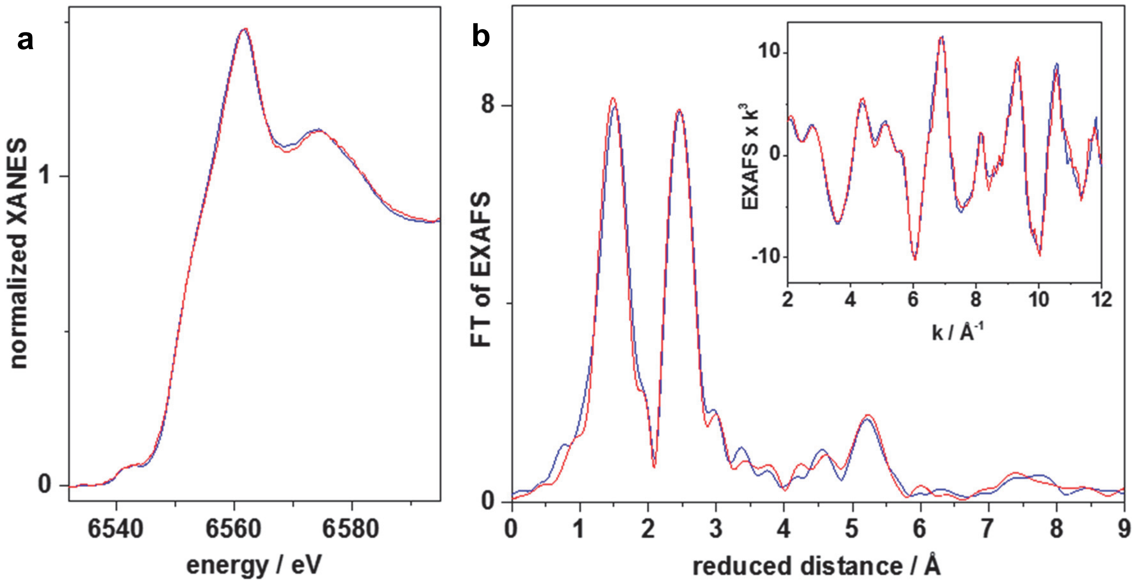

Supplementary Figure 15. Comparison of XAS data of Mn oxide in PSII at pH 7 (red lines) and 8.5 (blue lines). **(a)** XANES. **(b)** EXAFS. Data correspond to spectra in Figure 5 (pH 8.5, mean of data sets a and c) and Supplementary Figure 14 (pH 7, mean of data ±Ca). The comparison of the XANES and EXAFS spectra of the PSII-bound Mn-oxide formed at pH 7 and pH 8.5 does not reveal any significant differences.

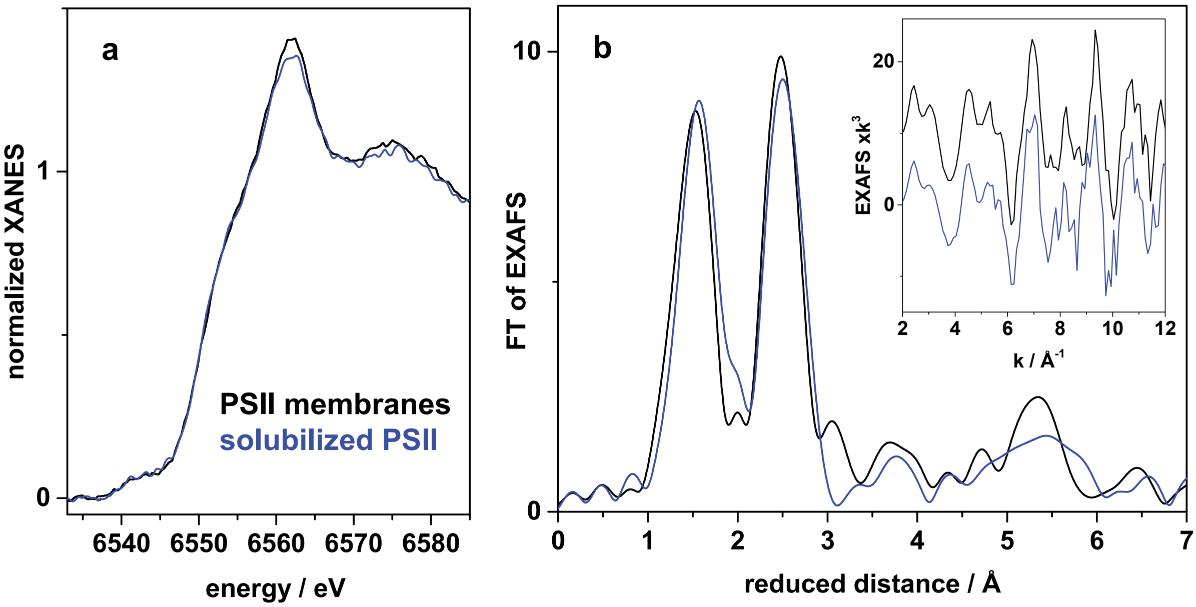

Supplementary Figure 16: XAS data of PSII membrane particles and solubilized PSII. **(a)** XANES spectra. **(b)** Fourier-transforms of EXAFS spectra in the inset. The Mn-depleted PSII preparations were illuminated in the presence of 30 µM PPBQ and 240 µM $MnCl_2$ in a calcium-containing buffer (pH 7). Black lines: PSII membrane particle preparation that was pelleted by centrifugation after the illumination. Blue lines: PSII membrane particles that were first solubilized in the illumination vessel by the addition of detergent and precipitated by polyethylenglycole (PEG) addition after the illumination. Both preparations were immediately loaded into sample holders for XAS and frozen in liquid nitrogen. For further details see the Materials and Methods and SI text.

Supplementary Table 1: Metal contents from TXRF in PSII preparations.[a]

preparation	Mn [mg/L] [μM]	Fe [mg/L] [μM]	Ca [mg/L] [μM]	Mn/PSII	Fe/PSII	Ca/PSII
(a) intact PSII	1.2±0.2<br>22±4	2.6±0.4<br>47±7	240.5±15<br>6000±380	4±1	9±2	1200±80
(b) Mn-depleted PSII	0.05±0.05<br><1±1	1.7±0.3<br>30±6	216.4±14<br>5400±350	<0.2±0.2	6±2	1080±70
(c) Mn-depl. PSII +240 μM Mn, dark	2.0±0.4<br>36±7	1.4±0.4<br>25±7	88.2±6.0<br>2200±150	7±2	5±1	440±30
(d) Mn-depl. PSII +240 μM Mn, light	18±5<br>330±90	1.4±0.4<br>25±7	92.2±8.0<br>2300±200	65±19	5±1	460±40

[a]Data represent mean values of three repetitions (±standard deviation) of TXRF measurements as shown in Supplementary Figure 1. The concentration of the Ga standard was 1 mg/L (14.3 μM), the chlorophyll (chl) concentration was 1 mg/mL (~1 mM), Mn/PSII, Fe/PSII, and Ca/PSII values were calculated using the earlier determined value of about 200 chlorophylls per PSII reaction center [1,2]. Note that calcium concentrations and Ca/PSII values reflect predominantly the calcium in the used buffer (5 mM $CaCl_2$) and calcium bound unspecifically to the membrane proteins. In addition, concentration determination in comparison to the gallium standard is less accurate at the relatively low energy of the Ca emission lines. According to the XAS data (Figure 5), PSII samples of type (c) contained about equal amounts of Mn(III) and Mn(IV) ions. Each PSII center therefore has transferred about 100 electrons, which would correspond to the double-reduction of about 50 DCPIP molecules per PSII. For ~0.1 μM PSII reaction centers (at ~20 μM chl) in the UV/vis assay of DCPIP (60 μM) reduction, the double-reduction of ~5 μM DCPIP, corresponding to a close to 10 % decrease of its absorption maximum at 604 nm thus was expected. This expectation is in reasonable agreement with the observed rapid decrease by ~0.1 units of the initial DCPIP absorption of ~0.8 (taking into account the chlorophyll background absorption) as shown in Figure 3.

Supplementary Table 2: EXAFS fit parameters.[a]

shell	Mn-O			Mn-Mn			
sample	N [per Mn]	R [Å]	$2\sigma^2$ x10^3 [$Å^2$]	N [per Mn]	R [Å]	$2\sigma^2$ x10^3 [$Å^2$]	R_F [%]
$Mn^{II}Cl_2$	$6^{\S}$	2.19	10	-	-	-	36.0
$Mn^{II}O$	$6^{\S}$	2.19 (2.22)	8	$12^{\S}$	3.12 (3.14)	6	27.4
				$6^{\S}$	4.50 (4.44)		
				$24^{\S}$	5.42 (5.44)		
				$12^{\S}$	6.39 (6.28)		
α-$Mn^{III}_2O_3$	$4.4^{\#}$	1.92 (1.96)	10	$6^{\S}$	3.10 (3.11)	12	17.8
	$1.6^{\#}$	2.22 (2.24)		$6^{\S}$	3.56 (3.58)	17	
				$6^{\S}$	4.70 (4.73)	8	
β-$Mn^{IV}O_2$	$6^{\S}$	1.87 (1.89)	7	$2^{\S}$	2.85 (2.88)	7	32.1
				$8^{\S}$	3.43 (3.43)		
				5.1	5.04 (5.07)		
birnessite $Mn^{III,IV}$	$5.0^{\#}$	1.90	4	4.8	2.89	9	11.9
				0.3	3.42		
	$1.0^{\#}$	2.30		2.2	5.01		
				2.4	5.52		
electrodeposited inactive MnOx	$4.9^{\#}$	1.90	5	4.8	2.86	7	18.1
				2.3	3.51		
	$1.1^{\#}$	2.33		2.5	5.02		
				6.0	5.55		
electrodeposited active MnOx (MnCat)	$5.1^{\#}$	1.89	8	2.3	2.87	9	15.0
				1.6	3.45		
	$0.9^{\#}$	2.30		1.4	5.01		
				1.7	5.48		
intact PSII (S_1)	$3.6^{\#}$	1.83	12	1.5	2.73	4	16.8
	$2.4^{\#}$	2.01		0.6	3.25		

Mn-depleted PSII (+3 min light, +240 µM $MnCl_2$, 5 mM $CaCl_2$, 60 µM PPBQ, pH 8.5)

(a)	4.7$^{\#}$	1.90	6	3.4	2.87	6	21.5
				0.7	3.46		
	1.3$^{\#}$	2.25		1.9	5.01		
				4.9	5.53		
(b)	4.6$^{\#}$	1.91	5	2.7	2.87	6	25.6
				0.8	3.45		
	1.4$^{\#}$	2.22		1.2	4.97		
				4.2	5.52		
(c)	5.2$^{\#}$	1.90	6	4.1	2.89	5	12.8
				0.8	3.54		
	0.8$^{\#}$	2.28		1.9	4.99		
				4.6	5.57		
mean	5.0$^{\#}$	1.90	6	3.5	2.87	6	13.5
				0.7	3.49		
	1.0$^{\#}$	2.25		1.5	4.99		
				4.2	5.54		

Mn-depleted PSII (+3 min light, +240 µM $MnCl_2$, with/without $CaCl_2$, PPBQ, pH 7.0)

shell	Mn-O			Mn-Mn			
sample	N [per Mn]	R [Å]	$2\sigma^2$ x10^3 [Å^2]	N [per Mn]	R [Å]	$2\sigma^2$ x10^3 [Å^2]	R_F [%]
Mn-depleted PSII (+1 min light, +240 µM $MnCl_2$, +30 µM PPBQ + 5 mM $CaCl_2$)	4.8$^{\#}$	1.89	8	3.5	2.86	4	18.4
				0.6	3.52		
	1.2$^{\#}$	2.24		1.7	5.01		
				4.6	5.56		
Mn-depleted PSII (+1 min light, +240 µM $MnCl_2$, +30 µM PPBQ - $CaCl_2$)	4.9$^{\#}$	1.89	7	3.5	2.87	5	12.0
				1.1	3.52		
	1.1$^{\#}$	2.28		1.4	4.98		
				4.2	5.56		

^{a}*N*, coordination number; *R*, interatomic distance; $2\sigma^2$, Debye-Waller parameter; R_F, fit error sum (for reduced distances of 1-5 Å). Fit restraints: §fixed parameter, $^{\#}$*N*-values coupled to yield a sum of 6. $^{\$}$These distances in intact PSII contain contributions from Mn-Ca vectors in the Mn_4CaO_5 complex. Parameters correspond to spectra in Figure 5 and Supplementary Figure 14. $MnCl_2$ was measured as 10 mM aqueous solution. For MnO, α-Mn_2O_3 and β-MnO_2 spectra commercial compounds were used and mixed with BN in ~1:20 ratio (supplier and purity on trace metal basis: Mn_2O_3 Sigma-Aldrich, 99.9%; MnO_2, Carl Roth, 99.995%; MnO, Sigma-Aldrich, 99%). The fit parameters for the Mn oxides comply with earlier EXAFS results on the respective and related species [17-22]. In parentheses, (average) distances determined by crystallography are given for $Mn^{II}O$ [23] (Crystallography Open Database ID 1514105), α-$Mn^{III}{}_2O_3$ [24] (Crystallography Open Database ID 1514103), and β-$Mn^{IV}O_2$ [25] (Crystallography Open Database ID 1514117). Good agreement between crystallographic and EXAFS distances verifies the intactness of the commercial Mn oxides. The birnessite spectrum corresponds to the $K_{0.31}$-birnessite spectrum published in [21], where further characterization of the material is provided. The spectra of electrodeposited active and inactive MnOx correspond to the spectra measured under open circuit conditions and published previously in [19].

Supplementary Note

Background on Mn^{2+} oxidation by PSII and hypotheses on the evolution of the oxygen-evolving complex (OEC) of PSII

Mn^{2+} oxidation by PSII: The characteristics of electron donation to PSII by Mn^{2+} are very well studied, but to our knowledge, an analysis of the structural and chemical configuration of the Mn products of the reaction, except in the case of photoactivation and the reformation of the native water-oxidizing Mn cluster, has not been described until now. Early studies on the properties of electron transport in the PSII reaction center showed that Mn^{2+} served as an electron donor to Mn-depleted PSII [10,26-29]. Of particular relevance, several studies showed that added Mn^{2+} could support sustained DCPIP photoreduction in Mn-depleted chloroplast and PSII preparations [10]. Hoganson and Babcock later showed Mn^{2+} was directly oxidized by the redox active tyrosine, Y_Z, of the D1 polypeptide [11] providing the basis for the current structural understanding of the pathway. Kinetic analysis of the competition between Mn^{2+} and other donors has subsequently provided further information on the affinity characteristics of Mn^{2+} binding [8,27,30]. While advancing the understanding of the binding of Mn ions to PSII, these various studies did not, however, provide information on the physiochemical nature of the products of the Mn^{2+} photochemical oxidation reaction as reported here. These studies did, however, lead to the discovery of photoactivation, which is the photochemical synthesis of the catalytically active Mn_4CaO_5 [31-35]. Unlike the very large arrays of Mn oxides described in the present work, photoactivation results in the formation of only the Mn_4CaO_5. Although much remains to be learned about photoactivation, important information on the nature of the structural intermediates has been published including EPR characterizations of early assembly intermediates [36,37].

The pioneering studies of George Cheniae showed that when photoactivation is performed under sub-optimal conditions, such as in the absence of Ca^{2+}, inactive Mn is accumulated. Under these conditions, the yield of Mn clusters that were capable of water-splitting decreased, yet Mn was found to be tightly bound in an EDTA unextractable, EPR silent form [38]. Cheniae referred to this form of photochemically assembled, but catalytically inactive Mn as ”inappropriately bound ligation of $Mn^{\geq 3+}$” [38] and the formation of these deposits corresponded to a type of photoinactivation preventing proper cluster assembly. However, these scientists did not characterize the form of the metal beyond estimating the number of atoms per reaction center nor did they speculate on whether or not these corresponded to Mn oxides. In retrospect, it appears likely that they were indeed making the Mn oxides in their reactions, although their comparatively small size would have made the structural characterization more difficult than what is now observed. It is worth noting that the Mn oxide structures considered by Russell and Hall [39] as well as by Sauer and Yachandra [40] were argued as abiotically formed and subsequently incorporated into a reaction center that was a precursor to modern PSII, which contrasts with the alternative proposal that the formation of the oxides occurs via photooxidation of Mn^{2+} at a reaction center.

Hypotheses on the evolution of the OEC: The evolutionary transition from anoxygenic photosynthesis to oxygenic photosynthesis would have involved the acquisition of metal binding features on the donor side of primitive anoxygenic photosynthetic reaction centers. Allen and co-workers have tested the hypothesis that it is possible to modify the redox and metal binding properties of anoxygenic purple bacterial reaction centers to produce modified complexes capable of oxidizing Mn^{2+} [41] Blankenship and Hartman proposed that the ancestral proto-PSII interacted with a Mn catalase [42]. Another class of proposals is that the Mn_4CaO_5 originates by the incorporation of a preformed mineral for the origin of a ‘preformed’ abiotic core cluster. Dismukes, Klimov and colleagues proposed that reaction center proteins acquired an O_2-evolving Mn cluster by binding of a tetramanganese-bicarbonate cluster derived

from the Mn-bicarbonate clusters present in the environment [43]. Russell and Hall performed seminal comparative analyses of the O_2-evolving Mn_4CaO_5 with naturally occurring inorganic Mn oxides [39]. They suggested photochemically formed MnO_2 precipitates in the early ocean were recruited and later modified to the O_2-evolving Mn cluster. They reasoned that the O_2-evolving Mn cluster may have derived from a coating of manganese precipitates containing Mn^{4+} that may have served as an electron acceptor and/or to protect from hard ultraviolet damage and "Thus, a cluster of ranciéite may have contributed the "ready-made" [$CaMn_4$] structure that was co-opted by one of the reaction centers, though if so the Mn-Mn distances of 2.9Å characteristic of the cluster constrained in ranciéite must have been modified to a conformation more typical of hollandite where two Mn-Mn distances are 2.7Å and two are 3.3Å" [39]. Sauer and Yachandra postulated that MnO was incorporated and gave a 'catalase' activity, releasing hydrogen peroxide [40]. Later, the pre-PSII complex was modified to utilize and assemble water-splitting Mn clusters: "The evolution of the modern complex, however, presumably began in the absence of the refined PS II protein binding site and fully developed mechanism for photo-oxidizing Mn^{2+} during its incorporation. It is well known that solid MnO_2 exhibits pronounced ''catalase'' activity in its ability to increase the rate of decomposition of H_2O_2 into H_2O and O_2 by many orders of magnitude." This falls into a class of hypotheses that conjecture that Mn oxides available in the environment were recruited into the ancient photochemical reaction center and subsequently modified.

Another class of proposals for the evolutionary origin of the O_2-evolving Mn_4CaO_5 hypothesizes that the metal cluster is a modification of metabolic adaptation of ancient microbial photosynthetic bacteria that utilized metals, first Fe^{3+} and later Mn^{2+}, as a source of metabolic reductant. Excellent recent synopsis on the current hypotheses regarding the evolution of photosynthetic reaction centers from a physiological perspective can be found in [44]. Zubay [45] and subsequently, Fischer et al. [46] proposed that an early precursor of PSII utilized solvated Mn^{2+} as a source of electrons for metabolism, hypothesizing that the resultant by-products were Mn oxides. These were hypothesized to be an intermediate to the evolution to a modified form of the Mn oxide that was catalytic and had become capable of supplying electrons from a secondary donor such as water. Fischer went on to assign this process to the origin of certain geologic Mn deposits, but his studies were focused more on the geologic processes and did not obtain evidence for hypothesized Mn oxide formation by photochemical reaction centers [46]. Thus, the formation of oxides by PSII, though hypothesized had not been shown before and that is the experimental novelty we now report.

Supplementary References

1 Barra, M. *et al.* Intermediates in assembly by photoactivation after thermally accelerated disassembly of the manganese complex of photosynthetic water oxidation. *Biochemistry* **45**, 14523-14532 (2006).

2 Barra, M., Haumann, M. & Dau, H. Specific loss of the extrinsic 18 kDa protein from photosystem II upon heating to 47 °C causes inactivation of oxygen evolution likely due to Ca release from the Mn-complex. *Photosynth. Res.* **84**, 231-237 (2005).

3 Allakhverdiev, S. I. *et al.* Reconstitution of the water-oxidizing complex in manganese-depleted photosystem II complexes by using synthetic binuclear manganese complexes. *Biochemistry* **33**, 12210-12214 (1994).

4 Dau, H. Molecular mechanisms and quantitative models of variable photosystem II fluorescence. *Photochem.Photobiol.* **60**, 1-23 (1994).

5 Krivanek, R., Kern, J., Zouni, A., Dau, H. & Haumann, M. Spare quinones in the Q_B cavity of crystallized photosystem II from Thermosynechococcus elongatus. *Biochim. Biophys. Acta* **1767**, 520-527 (2007).

6 Ono, T. A. & Mino, H. Unique binding site for Mn^{2+} ion responsible for reducing an oxidized Y_Z tyrosine in manganese-depleted photosystem II membranes. *Biochemistry* **38**, 8778-8785 (1999).

7 Semin, B. K. & Seibert, M. A carboxylic residue at the high-affinity, Mn-binding site participates in the binding of iron cations that block the site. *Biochim. Biophys. Acta* **1757**, 189-197 (2006).

8 Hsu, B. D., Lee, J. Y. & Pan, R. L. The high-affinity binding-site for manganese on the oxidizing side of photosystem-II. *Biochim. Biophys. Acta* **890**, 89-96 (1987).

9 Nixon, P. J. & Diner, B. A. Aspartate 170 of the photosystem II reaction center polypeptide D1 is involved in the assembly of the oxygen-evolving manganese cluster. *Biochemistry* **31**, 942-948 (1992).

10 Klimov, V. V., Allakhverdiev, S. I., Shuvalov, V. A. & Krasnovsky, A. A. Effect of extraction and re-addition of manganese on light reactions of photosystem-II preparations. *FEBS Lett.* **148**, 307-312 (1982).

11 Hoganson, C. W., Ghanotakis, D. F., Babcock, G. T. & Yocum, C. F. Mn(2+) reduces Y_z (+) in manganese-depleted photosystem II preparations. *Photosynth. Res.* **22**, 285-293 (1989).

12 Magnuson, A. & Andréasson, L.-E. Different manganese binding sites in photosystem II probed by selective chemical modification of histidyl and carboxylic acid residues. *Biochemistry* **36**, 3254-3261 (1997).

13 McKenna, J. M. & Bishop, N. I. Studies on the photooxidation of manganese by isolated chloroplasts. *Biochim. Biophys. Acta* **131**, 339-349 (1967).

14 Itoh, M., Yamashita, K., Nishi, T., Konishi, K. & Shibata, K. The site of manganese function in photosynthetic electron transport system. *Biochim. Biophys. Acta* **180**, 509-519 (1969).

15 Pospisil, P. Production of reactive oxygen species by photosystem II. *Biochim. Biophys. Acta* **1787**, 1151-1160 (2009).

16 Dittmer, J. *et al. Photosynthesis: Mechanisms and Effects* (Kluwer Academic Publishers, Dortrecht, 1998).

17 Frey, C. E. *et al.* Evaporated manganese films as a starting point for the preparation of thin-layer MnOx water-oxidation anodes. *Sustain. Energ.Fuels* **1**, 1162-1170 (2017).

18 González-Flores, D. *et al.* Electrosynthesis of biomimetic manganese-calcium oxides for water oxidation catalysis - atomic structure and functionality. *ChemSusChem* **9**, 379-387 (2016).

19 Mattioli, G., Zaharieva, I., Dau, H. & Guidoni, L. Atomistic texture of amorphous manganese oxides for electrochemical water splitting revealed by *ab initio* calculations combined with X-ray spectroscopy. *J. Am. Chem. Soc.* **137**, 10254-10267 (2015).

20 Zaharieva, I. *et al.* Electrosynthesis, functional and structural characterization of a water-oxidizing manganese oxide. *Energy Environ. Sci.* **5**, 7081-7089 (2012).

21 Wiechen, M., Zaharieva, I., Dau, H. & Kurz, P. Layered manganese oxides for water-oxidation: alkaline earth cations influence catalytic activity in a photosystem II-like fashion. *Chem. Sci.* **3**, 2330-2339 (2012).
22 Zaharieva, I. *et al.* Synthetic manganese-calcium oxides mimic the water-oxidizing complex of photosynthesis functionally and structurally. *Energy Environ. Sci.* **4**, 2400-2408 (2011).
23 Kuriyama, M. & Hosoya, S. X-ray measurement of scattering factors of manganese and oxygen atoms in manganous oxide. *J.Phys. Soc.Jpn.* **17**, 1022-1029 (1962).
24 Norrestam, R., Ingri, N., Östlund, E., Bloom, G. & Hagen, G. alpha-Manganese(III) oxide - a C-type sesquioxide of orthorhombic symmetry. *Acta Chem. Scand.* **21**, 2871-2884 (1967).
25 Bolzan, A., Fong, C., Kennedy, B. & Howard, C. Powder neutron diffraction study of pyrolusite, β-MnO_2. *Austr. J. Chem.* **46**, 939-944 (1993).
26 Ahlbrink, R., Semin, B. K., Mulkidjanian, A. Y. & Junge, W. Photosystem II of peas: effects of added divalent cations of Mn, Fe, Mg, and Ca on two kinetic components of P(+)(680) reduction in Mn-depleted core particles. *Biochim. Biophys. Acta* **1506**, 117-126 (2001).
27 Semin, B. K. *et al.* The extrinsic PsbO protein modulates the oxidation/reduction rate of the exogenous Mn cation at the high-affinity Mn-binding site of Mn-depleted PSII membranes. *J. Bioenerg. Biomembr.* **47**, 361-367 (2015).
28 Ben-Hayyim, G. & Avron, M. Mn^{2+} as electron donor in isolated chloroplasts. *Biochim. Biophys. Acta* **205**, 86-94 (1970).
29 Izawa, S. Photoreduction of 2,6-dichlorophenolindophenol by chloroplasts with exogenous Mn^{2+} as electron donor. *Biochim. Biophys. Acta* **197**, 328-331 (1970).
30 Semin, B. K. *et al.* Iron-blocking the high-affinity Mn-binding site in photosystem II facilitates identification of the type of hydrogen bond participating in proton-coupled electron transport via YZ. *Biochemistry* **44**, 9746-9757 (2005).
31 Cheniae, G. M. & Martin, I. F. Sites of function of manganese within photosystem II. Roles in O_2 evolution and system II. *Biochim. Biophys. Acta* **197**, 219-239 (1970).
32 Tamura, N. & Cheniae, G. Photoactivation of the water-oxidizing complex in photosystem-II membranes depleted of Mn and extrinsic proteins .I. biochemical and kinetic characterization. *Biochim. Biophys. Acta* **890**, 179-194 (1987).
33 Radmer, R. & Cheniae, G. M. Photoactivation of the manganese catalyst of 0_2 evolution II. A two-quantum mechanism. *Biochim. Biophys. Acta* **253**, 182-186 (1971).
34 Miller, A. F. & Brudvig, G. W. Manganese and calcium requirements for reconstitution of oxygen-evolution activity in manganese-depleted photosystem II membranes. *Biochemistry* **28**, 8181-8190 (1989).
35 Ono, T. A. & Inoue, Y. Photoactivation of the water-oxidation system in isolated intact chloroplasts prepared from wheat leaves grown under intermittent flash illumination. *Plant Physiol.* **69**, 1418-1422 (1982).
36 Campbell, K. A. *et al.* Dual-mode EPR detects the initial intermediate in photoassembly of the photosystem II Mn cluster: The influence of amino acid residue 170 of the D1 polypeptide on Mn coordination. *J. Am. Chem. Soc.* **122**, 3754-3761 (2000).
37 Tyryshkin, A. M. *et al.* Spectroscopic evidence for Ca^{2+} involvement in the assembly of the Mn_4Ca cluster in the photosynthetic water-oxidizing complex. *Biochemistry* **45**, 12876-12889 (2006).
38 Chen, C., Kazimir, J. & Cheniae, G. M. Calcium modulates the photo-assembly of photosystem II $(Mn)_4$-clusters by preventing ligation of nonfunctional high-valency states of manganese. *Biochemistry* **34**, 13511-13526 (1995).
39 Russell, M. J., Hall, A. J. & Mellersh, A. R. *Natural and Laboratory-Simulated Thermal Geochemical Processes* (Springer, Dordrecht, 2003).
40 Sauer, K. & Yachandra, V. K. A possible evolutionary origin for the Mn_4 cluster of the photosynthetic water oxidation complex from natural MnO_2 precipitates in the early ocean. *Proc. Natl. Acad. Sci. U.S.A.* **99**, 8631-8636 (2002).
41 Allen, J. P. & Williams, J. C. The evolutionary pathway from anoxygenic to oxygenic photosynthesis examined by comparison of the properties of photosystem II and bacterial reaction centers. *Photosynth. Res.* **107**, 59-69 (2011).
42 Blankenship, R. E. & Hartman, H. The origin and evolution of oxygenic photosynthesis. *Trends Biochem. Sci.* **23**, 94-97 (1998).

43 Dismukes, G. C. *et al.* The origin of atmospheric oxygen on Earth the innovation of oxygenic photosynthesis. *Proc. Natl. Acad. Sci. U.S.A.* **98**, 2170-2175 (2001).
44 Martin, W. F., Bryant, D. A. & Beatty, J. T. A physiological perspective on the origin and evolution of photosynthesis. *FEMS Microbiol. Rev.* **42**, 205-231 (2018).
45 Zubay, G. *Origins of Life on Earth and in the Cosmos*. (Academic Press, 1996).
46 Johnson, J. E. *et al.* Manganese-oxidizing photosynthesis before the rise of cyanobacteria. *Proc. Natl. Acad. Sci. U.S.A.* **110**, 11238-11243 (2013).
