# Supplementary material for: Light-driven formation of manganese oxide by today’s photosystem II supports evolutionarily ancient manganese-oxidizing photosynthesis
Source: Nat Commun. 2020 Nov 30;11:6110. doi: 10.1038/s41467-020-19852-0 (PMC7705724; doi:10.1038/s41467-020-19852-0)
Supplement: Supplementary file 2 — Reporting Summary [file 41467_2020_19852_MOESM2_ESM.pdf]

nature research

Corresponding author(s): Holger Dau

Last updated by author(s): Oct 1, 2020

Reporting Summary

Nature Research wishes to improve the reproducibility of the work that we publish. This form provides structure for consistency and transparency in reporting. For further information on Nature Research policies, see our Editorial Policies and the Editorial Policy Checklist.

Statistics

For all statistical analyses, confirm that the following items are present in the figure legend, table legend, main text, or Methods section.

n/a	Confirmed	
☐	☒	The exact sample size (n) for each experimental group/condition, given as a discrete number and unit of measurement
☐	☒	A statement on whether measurements were taken from distinct samples or whether the same sample was measured repeatedly
☒	☐	The statistical test(s) used AND whether they are one- or two-sided<br>*Only common tests should be described solely by name; describe more complex techniques in the Methods section.*
☒	☐	A description of all covariates tested
☒	☐	A description of any assumptions or corrections, such as tests of normality and adjustment for multiple comparisons
☒	☐	A full description of the statistical parameters including central tendency (e.g. means) or other basic estimates (e.g. regression coefficient) AND variation (e.g. standard deviation) or associated estimates of uncertainty (e.g. confidence intervals)
☒	☐	For null hypothesis testing, the test statistic (e.g. F, t, r) with confidence intervals, effect sizes, degrees of freedom and P value noted<br>*Give P values as exact values whenever suitable.*
☒	☐	For Bayesian analysis, information on the choice of priors and Markov chain Monte Carlo settings
☒	☐	For hierarchical and complex designs, identification of the appropriate level for tests and full reporting of outcomes
☒	☐	Estimates of effect sizes (e.g. Cohen's d, Pearson's r), indicating how they were calculated

Our web collection on statistics for biologists contains articles on many of the points above.

Software and code

Policy information about availability of computer code

Data collection	Inhouse software for X-ray data collection (writen by Dr. Petko Chernev, coauthor): KMC-3-suite 2020<br>The code allows for XAS data averaging and primary analysis using standard methodology. The specific code is irrelevant for our conclusions.
Data analysis	Inhouse software for X-ray data analysis (writen by Dr. Petko Chernev, coauthor): SimXlite 13.0, Bessy 43.0<br>The code allows for XAS data analysis using standard methodology. The specific code is irrelevant for our conclusions.<br><br>Commercial software: OriginPro 2016G, Microsoft Excel 2013

For manuscripts utilizing custom algorithms or software that are central to the research but not yet described in published literature, software must be made available to editors and reviewers. We strongly encourage code deposition in a community repository (e.g. GitHub). See the Nature Research guidelines for submitting code & software for further information.

Data

Policy information about availability of data

All manuscripts must include a data availability statement. This statement should provide the following information, where applicable:

- Accession codes, unique identifiers, or web links for publicly available datasets
- A list of figures that have associated raw data
- A description of any restrictions on data availability

All data needed to support the conclusions of this manuscript are included in the main text and SI Appendix. The source data underlying Figs. 3, 4 and 5 are provided as a Source Data file.

Field-specific reporting

Please select the one below that is the best fit for your research. If you are not sure, read the appropriate sections before making your selection.

- [x] Life sciences
- [ ] Behavioural & social sciences
- [ ] Ecological, evolutionary & environmental sciences

For a reference copy of the document with all sections, see nature.com/documents/nr-reporting-summary-flat.pdf

Life sciences study design

All studies must disclose on these points even when the disclosure is negative.

Sample size	Sample sizes were not predetermined based on specific statistical methods, but were chosen according to the standards of the field (for moderate deviations between data sets, minimally three independent replicates for each condition).<br>The number of spectral scans averaged to obtain a single, subsequently analyzed spectrum were chosen such that the averaged spectrum exhibits an appropriately high signal-to-noise ratio; visual inspection of the shown spectra allows for verification of a good signal-to-noise ratio.
Data exclusions	For the X—ray data collected at the synchrotron radiation source (BE55Y), data that was affected by obvious technical problems (aborted scans, misadjusted X—ray beam, etc.) was excluded from further the analysis. At a later stage, only the X-ray data that was unrelated to our conclusions was not selected for presentation.<br>For the UV—vis data, all experiments have been repeated several times. As there were no indications for irreproducible aspects that could matter for our conclusions, typical data is shown.<br>For the SDS—gel photo in Supplementary Figure 2, we selected the data that was best suited for visual inspection; other SDS—gel data did not show any diverging behavior. For the variable—fluorescence data of Supplementary Figure 9, panel a and panel b show illustrative typical data; all data is shown for the parameter displayed in panel c.
Replication	For the central X—ray data, reproducibility is verified by presenting and analyzing three data sets (P5II—MnOx (a), (b), and (c) in Figure 5 and Supplementary Table 2). The data in Supplementary Figures 14, 15, and 16 ensured the absence of a calcium influence, pH influence, or solubilization influence; here only a single data set was collected for each experimental condition.<br>For the UV—vis data, all experiments were repeated at least 3 times. As there were no indications for irreproducible aspects that could matter for our conclusions, typical data is shown.<br>For the SDS—gel photo in Supplementary Figure 2, we selected the data that was best suited for visual inspection; 3 replicates were performed and other SDS—gel data did not show any diverging behavior.<br>For the variable—fluorescence data of Supplementary Figure 9, panel a and panel b show illustrative typical data from 3 data sets; all collected data is shown for the parameter displayed in panel c.<br>All experimental replicates for the above methods were successful and gave similar results.
Randomization	Randomization is not applicable, as we measured physical values, which are not influenced by the observer. Randomization is not typically used in the field.
Blinding	Blinding is not applicable, because we did not study the behavior or response of a group of study participants. Blinding of experimentators was not necessary, because of fully automated (computerized) data collection, which excludes that the collected data is affected by a subjective bias of the experimentator. Blinding is not typically used in the field.

Reporting for specific materials, systems and methods

We require information from authors about some types of materials, experimental systems and methods used in many studies. Here, indicate whether each material, system or method listed is relevant to your study. If you are not sure if a list item applies to your research, read the appropriate section before selecting a response.

Materials & experimental systems

n/a	Involved in the study	
☒	☐	Antibodies
☒	☐	Eukaryotic cell lines
☒	☐	Palaeontology and archaeology
☒	☐	Animals and other organisms
☒	☐	Human research participants
☒	☐	Clinical data
☒	☐	Dual use research of concern

Methods

n/a	Involved in the study	
☒	☐	ChIP-seq
☒	☐	Flow cytometry
☒	☐	MRI-based neuroimaging
